# Electronic Health Record–Based Decision-Making Support in Inpatient Diabetes Management

**DOI:** 10.1007/s11892-022-01481-0

**Published:** 2022-08-02

**Authors:** Johanna E. Gerwer, Grace Bacani, Patricia S. Juang, Kristen Kulasa

**Affiliations:** Department of Internal Medicine, Division of Endocrinology and Metabolism, University of California San Diego, San Diego, CA USA

**Keywords:** Electronic health record, Clinical decision support, Order sets, Inpatient diabetes management, Hyperglycemia

## Abstract

**Purpose of Review:**

This review discusses ways in which the electronic health record (EHR) can offer clinical decision support (CDS) tools for management of inpatient diabetes and hyperglycemia.

**Recent Findings:**

The use of electronic order sets can help providers order comprehensive basal bolus insulin regimens that are consistent with current guidelines. Order sets have been shown to reduce insulin errors and hypoglycemia rates. They can also help set glycemic targets, give hemoglobin A1C reminders, guide weight-based dosing, and match insulin regimen to nutritional profile. Glycemic management dashboards allow multiple variables affecting blood glucose to be shown in a single view, which allows for efficient evaluation of glucose trends and adjustment of insulin regimen. With the use glycemic management dashboards, active surveillance and remote management also become feasible. Hypoglycemia prevention and management are another part of inpatient diabetes management that is enhanced by EHR CDS tools. Furthermore, diagnosis and management of diabetic ketoacidosis and hyperglycemia hyperosmolar state are improved with the aid of EHR CDS tools.

**Summary:**

The use of EHR CDS tools helps improve the care of patients with diabetes and hyperglycemia in the inpatient hospital setting.

## Introduction

Inpatient glycemic control is an important issue, as hyperglycemia, hypoglycemia, and glucose variability in the hospital setting are associated with adverse outcomes including higher in-hospital mortality rates, greater use of healthcare resources, and increased hospital complications, including higher infection rates, delayed wound healing, prolonged surgical recovery, and extended hospital stays [1–4]. Over 10% of the US population carries a diagnosis of diabetes, but in the hospital setting, up to 40% of patients are known to have diabetes or hyperglycemia [5, 6]. Careful glycemic management aimed at maintaining optimal blood glucose levels can lessen some of the attendant financial and non-financial burdens that have been observed when there is suboptimal glycemic control in the hospital setting [6, 7].

One effective approach to reducing complications resulting from suboptimal glycemic control for patients with diabetes in the inpatient setting has been the use of clinical decision-making support (CDS) tools, which merge patient data with population statistic and best-practice guidelines, in real time, in the electronic health record (EHR). Multiple guiding endocrine organizations, including American Diabetes Association (ADA) and American Association of Clinical Endocrinology (AACE), have advocated for the use of EHR CDS tools embedded into protocols, algorithms, and order sets to improve diabetes care in the inpatient setting [3, 8]. In this article, we address the use of EHR CDS tools in the management of inpatient diabetes, with a particular focus on electronic order sets, glycemic management dashboard, remote surveillance, and protocol use for specific scenarios.

## Order Sets

EHR CDS tools can utilize individual patient data joined with population statistics and best-practice guidelines to give or facilitate patient-specific recommendations [9]. These tools can be embedded in the EHR into current workflows and provide assistance to providers in real time to efficiently and effectively help guide decision-making. Computerized provider order entry (CPOE) order sets are one tool that can enhance the efficacy of EHR CDS tools [10]. CPOE order sets aggregate orders related to a specific management issue, such as a medical diagnosis of diabetes or use of insulin in the inpatient setting, facilitating succinct, effective, and efficient ordering of medications and treatments for patients in the hospital setting [10, 11]. Furthermore, CPOE order sets support best-practice recommendations to guide medical providers in real time utilizing prompts and alerts to order all relevant items to help improve meaningful use and quality metrics [4].

For inpatient glycemic control, these order sets support providers in ordering comprehensive basal bolus insulin regimens consistent with current guidelines [7]. CPOE order sets utilizing CDS have been shown to reduce insulin errors, hypoglycemic events [6, 12]. Their use has been advocated for by the National Academy of Medicine as a means of preventing medication errors and improving the efficacy of medication administration. Furthermore, multiple quality improvement organizations such as the Institute for Safe Medication Practices (ISMP) and guiding specialty organizations like the ADA support the use of order sets [3, 13]. ISMP has detailed guideline for the use of order sets and order set structure to allow order set implementation to be done effectively [13].

## Glycemic Targets

Blood glucose targets have been established for most hospitalized patients as between 140 and 180 mg/dL [4]. Notably, specific populations (e.g., post-cardiac surgery patients) have more stringent targets while others such as patients of advanced age with multiple comorbid conditions have more lenient targets [4, 14].

Glycemic targets are frequently not met in the inpatient setting, most commonly due to the inappropriate prescribing of insulin [6, 15]. The use of CDS in CPOE insulin order sets and glycemic management algorithms is an effective intervention that results in an increase in patients achieving blood glucose values in the desired range. In a study done by Maynard et al., the adoption of structured insulin order sets and insulin management algorithms, hypoglycemia rates were reduced from 3.8% patient days to 2.6% patient days, a relative-risk reduction of 0.68. Additionally, Maynard et al. found that the percent of uncontrolled patient days significantly decreased from 37.8 to 30.1% [16]. Furthermore, CDS systems are associated with a decrease in insulin dosing errors that can be further increased when CDS are incorporated into an EHR as electronic orders, rather than paper versions. In a post-hoc analysis by Donsa et al. on the impact of errors observed from paper-based versus computerized diabetes management in hospitalized patients with type 2 diabetes, an eightfold increase in the incidence of insulin dosing errors occurred in the paper-based group [17].

In addition, CPOE-based order sets can be used to allow for appropriate, safe, and efficient insulin orders to be placed by bundling all relevant orders together. More important components of a CPOE order set for an EHR CDS tool that addresses inpatient blood glucose based on a range of literature are shown in Table 1. Structured order sets can provide built-in decision support, permitting optimized, and comprehensive diabetes care [7].

**Table 1:** Summary of CPOE order set components for an EHR CDS tool that addresses inpatient blood glucose

Components of CPOE order set:	Examples:
Identify blood glucose target	Blood glucose targets 140 to 180 mg/dL Unique populations may have different targets (e.g., elderly or post-CABG patients)
POC glucose testing	Every 6 h for continuous nutrition Every meal and before bed for three times a day carbohydrate limited
HbA1c on admission	Updated HbA1c at hospital admission An HbA1c in the preceding two months is acceptable
Basal bolus insulin orders	Guidance to weight-based insulin dosing Recommendations for specific clinical scenarios (e.g., holding parameters for hypoglycemia) Prompts to select insulin type based on nutritional status Reminders to make dosing modifications based on factors that influence glycemia (e.g., renal function and steroid use)
Discontinue of non-insulin antihyperglycemic agents	On admission prompts to discontinue oral and non-insulin injectable medications, in most clinical circumstances
Diabetes education	Bedside nursing teaching, pharmacy medication education, dietitian nutritional guidance, instructional handouts, and diabetes basics tutorials

[6, 8, 16]

## Ordering HbA1c

A recently obtained HbA1c should be available and, if not, obtained, as it grossly indicates the level of glycemic control around the time of the admission [4, 18]. This information can be helpful when considering appropriate insulin dosing on admission as well adjusting outpatient diabetes medication management upon discharge [19]. An integrated CDS order set should include the recommendation to order an updated HbA1c if one has not resulted in the past 2–3 months. CDS within order sets can be structured to automatically default ordering an HbA1c if an HbA1c result is not present in the EHR in the preceding months [19].

## Weight-Based Dosing Guidance

The use of basal bolus insulin is strongly encouraged by multiple expert groups as it is more consistent with physiologic insulin secretion than a reactionary approach using sliding-scale insulin monotherapy [20, 21]. Furthermore, the use of basal bolus insulin allows for improved glycemic control and lowers total daily doses of insulin to achieve such control [3, 16, 21]. Weight-based insulin dosing estimates a patient’s anticipated insulin needs based on weight. However, factors beyond the patient’s weight might also affect those needs. For example, insulin needs of a patient may vary based on nutritional status, renal function, steroid use, the severity and nature of the illness that resulted in the hospital admission, and the type of diabetes [8]. Therefore, weight-based insulin dosing should be a guide for initial insulin dosing, supplemented by consideration of patient-specific factors and adjustments arising from changes that occur during the patient’s hospitalization [16].

The use of CDS in CPOE for weight-based insulin dosing allows for appropriate correctional insulin to be ordered in addition to basal and bolus insulin. Correction scales can be a standard scale or matched with a patient’s degree of insulin sensitivity. Scales that are reflective of an individual’s insulin sensitivity have demonstrated increased rates of blood glucose values in the optimal range, and CDS tools embedded within the CPOE order sets can help guide the provider in choosing an appropriate correction scale or correction factor [20]. In a study by Wong et al., a higher proportion of optimized blood glucose control was achieved in patients using order sets with sensitivity-based correctional insulin when compared to patients using order sets without sensitivity-based correctional insulin (65.3% vs. 55.0%, *p* < 0.001) [20].

## Insulin Regimens for Variable Nutritional Status

Among the many complex elements of inpatient care that impact glycemic control, nutritional status is one of the most significant [7]. The integration of CDS into order entry systems can guide providers to select the insulin type with the profile that is most appropriate for the patient’s nutritional status. The use of long-acting basal insulin is recommended in most situations. However, nutritional insulin depends on the nutrition being provided. For patients who are eating, rapid acting analogues are frequently used for nutritional coverage. Alternatively, patients receiving continuous enteral or parenteral nutrition might need a longer acting insulin or a different frequency of short acting insulin. Many institutions have developed CPOE order sets that utilize CDS to help match insulin type selection with nutritional status [16].

Furthermore, inclusion of standardized insulin administration instructions in the order set should be defaulted in an effort to prevent hypoglycemia. Changes or interruption in nutrition is one of the primary causes of hypoglycemia in the hospital [6, 22], so indication and holding parameters for nutritional insulin administration orders should be included in every nutritional insulin order. While basal insulin, if appropriately dosed, should not have to be held if a patient is temporarily NPO, holding parameters can also be specified for added hypoglycemia preventive measures [6]. Additionally, nutritional insulin orders can be written to allow for nutritional insulin doses to be modified consistent with a patient’s carbohydrate intake [16]. For example, after nursing education and appropriate process development, order sets can be used that better accommodate for uncertain nutritional intake by allowing nursing staff to delay nutritional insulin in a patient with inability to tolerate a meal or adjust the dose based on the percentage meal or amount of carbohydrate consumed [19].

## Notification Prompts

CPOE order sets with integrated CDS have demonstrated greater adoption of best practice recommendations [16, 20]. For example, the use of CPOE CDS order sets can discourage the inappropriate prescription of insulin by requiring providers to justify the use of insulin in a manner inconsistent with current recommendations [16]. Maynard et al. found that the percent of isolated sliding scale insulin ordering, deterred by guiding societies, dramatically decreased with the use of structured subcutaneous insulin order sets with built in CDS (72% to 26%, *P* < 0.0001) [16]. Additional work by Wong et al. found that the use of computerized insulin order sets increased the use of basal-bolus-correctional insulin (20.3% vs 23.6%, *P* < 0.0001) [20]. Embedded in CPOE order sets can be decision support cues to improve diabetes patient-management practices [23]. For example, on admission, with the initiation of an insulin order set, a reminder to discontinue all oral antihyperglycemic and non-insulin injectable medications can easily be integrated into the order set. Furthermore, their use in conjunction with insulin management algorithms can allow providers to select the insulin type, amount, and timing that is the most appropriate for the patient’s clinical situation.

EHR-based CDS systems can prompt providers to order diabetes education that can be offered through a variety of mechanisms such as e-learning platforms, pharmacists, certified diabetes educators, specialized teams, or general nursing staff. Diabetes education in the inpatient setting can improve outpatient management and hospital readmission rates [24]. Lower rates of hospital readmission 30 days after discharge were demonstrated at one hospital when inpatient diabetes education was delivered (11 vs 16%, *P* = 0.0001) [25]. CDS within order sets can support educational effort, for example, by embedding pre-checked orders for e-learning platforms or automatically adding diabetes patient education to RN work-queues.

## Glucose-Insulin Display

Once insulin orders are placed with the aid of order sets; the next task of managing and adjusting the treatment regimen can be cumbersome and complex. Assessment of glycemic control and treatment decisions must be performed in the context of a multitude of intertwined variables including nutritional status and intake, insulin type, dose, and timing, especially as it relates to nutrition, steroid administration, renal function, and clinical status. Examining changing variables and relating them temporally to blood glucose patterns in order to synthesize a treatment plan conventionally require navigation to multiple areas of the EHR. Gathering information from this scattered display is time-intensive, increases cognitive burden on the provider, and can lead to suboptimal decisions [26] that in turn presents patient safety concerns [27].

**Fig. 1:**
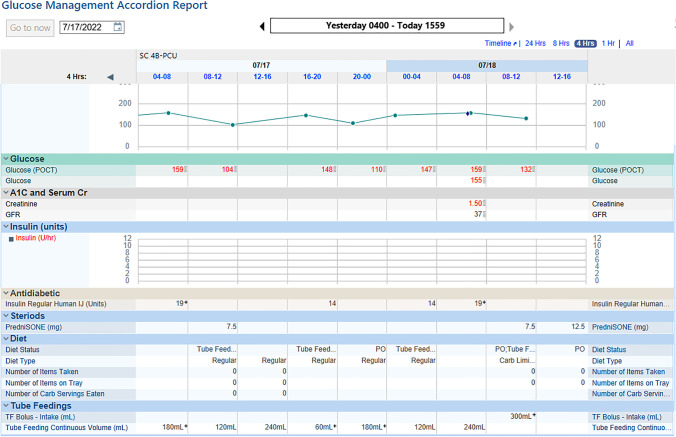
Glucose-insulin display. When available and in addition to items already displayed above, oral anti-diabetes medications, parenteral nutrition rate, and dextrose IV for hypoglycemia treatment will populate in this page

**Fig. 2:**
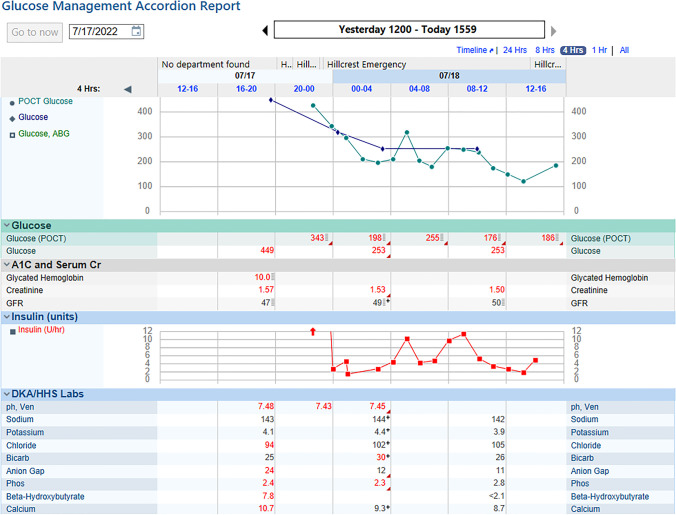
Glucose-insulin display. When available and in addition to items already displayed above, oral anti-diabetes medications, parenteral nutrition rate, and dextrose IV for hypoglycemia treatment will populate in this page

The EHR can be used to display information pertinent to formulate glycemic treatment recommendations in a succinct and intuitive manner. Many EHRs come equipped with a glucose-insulin display that is customizable and can be used effectively as one of the tools for blood glucose management. Glucose-insulin displays can show multiple variables that affect blood glucose in a single view, with particular attention to the timing of insulin and other glucose-affecting medications (i.e., steroids, oral DM medications), and most commonly contributing factors such as nutrition and renal function. As seen in Figs. 1 and 2, the glucose-insulin display in different EHR’s can lay out insulin types and doses, nutritional status and intake, steroid administration, and pertinent labs in a single view so they can be examined in real-time in relation to blood glucose readings and patterns. This type of display eliminates the need to gather multiple pieces of information from separate areas of the EHR and thereby allowing more informed and timely interventions. Historical data in the same admission can also be viewed on this display, allowing the clinician to quickly refer to prior glycemic patterns in view of the aforementioned variables.

## Active Surveillance

Active surveillance is another EHR tool to aid in blood glucose management in the hospital. The EHR can easily be queried to generate reports to identify patient cases with potential glycemia-related gaps in care. Institutions can define parameters at which to screen. For example, some hospitals generate hypoglycemia reports for blood glucose thresholds using < 80 mg/dL or < 70 mg/dL as the cutoff level, while others use < 50 mg/dL. Similarly, institutions can set different hyperglycemia thresholds to capture. The frequency of events can also be customized, as well the duration of look-back periods that can range from hours to days [6, 28]. Moreover, some have designed EHR tools that screen for both dysglycemia and inappropriate condition-specific treatment regimen. For instance, one institution designed a program that screened for severe (BG > 250 mg/dL at least once) or persistent hyperglycemia (> 180 mg/dL at least twice 3 h apart) in those with diabetes and stress hyperglycemia on only sliding scale insulin, and for type 1 diabetes on sliding scale insulin alone [29]. Once cases are found, interventions vary widely, from real-time EHR alerts to clinicians with clinical decision support tools, to communicating suggestions for treatment changes via paging system or note in the patient chart, to a full in-person endocrinology consult [15, 30–33]. The patient population, frequency, blood glucose cutoffs, look-back period, associated criteria, and plan for intervention are all customizable and are often dictated by the amount of resources available to assist in such efforts. Leveraging the EHR to generate the lists with a direct link to the patient chart can significantly reduce the amount of time needed for screening. This active surveillance coupled with real-time intervention, known as “measure-vention,” along with targeted and multifaceted interventions, has been found to lower hypo- and hyperglycemia rates in hospitalized patients [31–33].

## Remote Management

Other institutions have combined active surveillance and glucose-insulin displays to form the crux of a successful inpatient remote glucose management service [28, 34]. In one hospital, daily reports are automatically generated for patients with hyperglycemia and hypoglycemia defined as 2 or more readings of ≥ 225 mg/dL and < 70 mg/dL, respectively, on insulin pumps, and those with type 1 diabetes, in the preceding 24 h. From these reports, the glucose management service will then access individual patient glucose-insulin displays to assess glycemic patterns, as well as review the chart for orders for nutrition, insulin, steroids, and recent notes to determine any anticipated changes in patient treatment. A glucose management service note is then entered into the EHR for the primary team to view and act upon as applicable, with a disclaimer stating recommendations should be taken in light of a patient’s current clinical status and to obtain a formal endocrinology consult as necessary. This remote glucose management program resulted in 39% and 36% lower proportion of hospitalized patients with institution-defined hyperglycemia and hypoglycemia, respectively [28], and was well-accepted by providers [15]. Another institution implemented a similarly structured remote glucose management program and observed a 43% decrease in the rate of hyperglycemia (2 or more BG readings ≥ 300 mg/dL) and 50% decrease in rate of hypoglycemia (BG ≤ 70 mg/dL) [34].

## Hypoglycemia Prevention, Management, and Nursing Documentation

Another useful EHR feature in inpatient glycemic control is the ability to embed hypoglycemia prevention and management. For instance, predictive analytic tools can be utilized in the EHR to evoke real-time alerts for patients at high risk for hypoglycemia [35, 36]. One hospital observed a 68% decrease in severe hypoglycemia with integration of a predictive tool that alerted users of patients at high risk for low blood glucose [37].

Moreover, a hospital-wide, nurse-driven hypoglycemia protocol can also be default-selected when ordering insulin using the order set. This ensures all patients on insulin have a standardized hypoglycemia protocol ordered and ready for use. Having this protocol ordered automatically for all patients on insulin improves patient safety as it eliminates the time consuming task for the nurse to contact the provider and await orders to treat hypoglycemia. Nurse-initiated strategies to treat hypoglycemia can have slight differences between institutions but the aim is to provide 15 g of fast-acting carbohydrates, rechecking blood glucose in 15 min, and repeating the treatment and blood glucose check every 15 min until hypoglycemia is resolved [38]. With features like these, insulin order sets in the EHR that include standing orders for hypoglycemia treatment have been associated with reduced rates of hypoglycemia [16, 39].

Identifying the etiology of hypoglycemia in real time can also help reduce recurrent hypoglycemic events. The EHR can help support this process by standardizing hypoglycemia documentation and including prompts related to etiology. These prompts empower nurses to identify and report suspected causes of the hypoglycemia episode to the provider, in order for the provider to make treatment changes to prevent its recurrence. This documentation is also recommended by the ADA for each hypoglycemia event for this reason [3]. One institution observed a reduction in rates of inpatient hypoglycemia (BG < 70 mg/dL) from 2.3 to 1.5% (*P* < 0.001) and recurrent hypoglycemia (3 or more BG < 70 mg/dL during hospital stay) from 5.7 to 2.2% (*P* = 0.044) after instituting an automated tool that elicited from nurses the possible causes at the time of the hypoglycemia event [39].

## Diabetic Ketoacidosis and Hyperglycemic Hyperosmolar State Support

The EHR can also offer CDS in the management of hyperglycemic emergencies, such as diabetic ketoacidosis (DKA) and hyperglycemic hyperosmolar state (HHS). The diagnosis and management of DKA and HHS can be complex. It is important to arrive at the correct diagnosis because of the high cost and high mortality rate associated with DKA/HHS if not treated promptly and appropriately [40]. The EHR can provide CDS on appropriate diagnosis and management of DKA and HHS. Protocols should include guidance on the glycemic target ranges, frequency of blood glucose monitoring, insulin infusion dosing, and calculation of insulin sensitivity [19]. DKA and HHS order sets should provide guidance on initial intravenous fluid, when to change to dextrose containing fluid, potassium repletion, and frequency of electrolyte checks [40]. Hypoglycemia protocols should also be part of insulin infusion order sets, along with algorithms for nurses to follow in the event of interruption of insulin infusion or interruptions in nutritional intake [19]. Implementation of computerized DKA and HHS order sets and protocols has been shown to improve compliance with ADA DKA guidelines, 24-h fluid resuscitation, time to DKA resolution, and appropriate transition to subcutaneous insulin [41].

Computer-guided insulin infusion protocols have been shown to be as effective, if not superior, to standard column–based paper algorithms in the treatment of DKA and HHS [42, 43]. Ullal et al. showed that treatment of DKA with a computer-based algorithm was associated with lower hypoglycemia rates and faster time to resolution of DKA when compared to a paper form based–insulin infusion algorithm [43]. At our institution, we transitioned our insulin infusion computer calculator directly into the electronic medication administration record of our EHR. For patients admitted with hyperglycemic emergency, there was no difference between time in range between pre and post transition of our insulin infusion calculator. Nursing satisfaction was higher with the computer-based calculator, with 75 out of 79 responders favoring the computer calculator [44].

## Conclusion

Suboptimal blood glucose control in the hospital is associated with adverse outcomes, including higher mortality, increased risk for infections, greater financial costs, and longer lengths of stay. The use of CDS tools in the EHR has been found to increase provider adherence to standards of care, provide both guidance and safeguards in the provision of diabetes care, and improve inpatient glycemic control.
